# Simultaneous Stochastic Optimization of Mining Complexes and Mineral Value Chains

**DOI:** 10.1007/s11004-017-9680-3

**Published:** 2017-03-02

**Authors:** Ryan Goodfellow, Roussos Dimitrakopoulos

**Affiliations:** COSMO - Stochastic Mine Planning Laboratory, McGill University, 3450 University Street, Montreal, QC H3A 0E8 Canada

**Keywords:** Mining complex, Stochastic optimization, Metaheuristics, Materials mined and supply uncertainty, Stochastic or geostatistical simulation

## Abstract

Recent developments in modelling and optimization approaches for the production of mineral and energy resources have resulted in new simultaneous stochastic optimization frameworks and related digital technologies. A mining complex is a type of value chain whereby raw materials (minerals) extracted from various mineral deposits are transformed into a set of sellable products, using the available processing streams. The supply of materials extracted from a group of mines represents a major source of uncertainty in mining operations and mineral value chains. The simultaneous stochastic optimization of mining complexes, presented herein, aims to address major limitations of past approaches by modelling and optimizing several interrelated aspects of the mineral value chain in a single model. This single optimization model integrates material extraction from a set of sources along with their uncertainty, the related risk management, blending, stockpiling, non-linear transformations that occur in the available processing streams, the utilization of processing streams, and, finally, the transportation of products to customers. Uncertainty in materials extracted from the related mineral deposits of a mining complex is represented by a group of stochastic simulations. This paper presents a two-stage stochastic mixed integer nonlinear programming formulation for modelling and optimizing a mining complex, along with a metaheuristic-based solver that facilitates the practical optimization of exceptionally large mathematical formulations. The distinct advantages of the approach presented herein are demonstrated through two case studies, where the stochastic framework is compared to past approaches that ignore uncertainty. Results demonstrate major improvements in both meeting forecasted production targets and net present value. Concepts and methods presented in this paper for the simultaneous stochastic optimization for mining complexes may be adopted and applied to the optimization of smart oil fields.

## Introduction

**Fig. 1:**
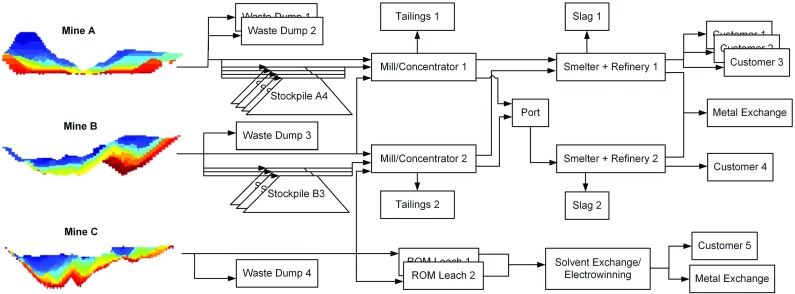
An example of a mining complex. Materials flow from the mines on the *left* to the products delivered to customers or the spot market in the far *right* of the figure

A mining complex is a mineral value chain where raw material flows from open pit and/or underground mines to the mineral markets after being treated and transformed into sellable products. Components of a mining complex include mineral deposits, stockpiles, waste disposal, processing destinations, utilization of processing capabilities, products and transportation systems (Fig. 1); all of which constitute a complex non-linear system. The primary objective is to define a production schedule that: (1) maximizes the net present value of the mining complex when products reach customers or the spot market; (2) ensures that technical constraints are obeyed; and (3) leads to a high likelihood that production targets are met, by accounting and managing technical risks due to the uncertainty in the spatial characterization of the pertinent properties of mineral deposits. This is referred to as geological uncertainty, and includes metal grades, material types, geometallurgical characteristics, volumes and spatial geometries of materials, and other matters. It should be noted that a mine’s production schedule is defined by the extraction sequence of the materials from the ground (e.g., mining blocks in an open pit or stopes from an underground mine), the destination policy decisions that define where extracted materials are sent, the processing stream decisions that define the quantities of materials sent from one destination to another in the value chain, and the utilization of processing capacities available. Examples of destinations that may be modelled in a mineral value chain include stockpiles, crushers, mills, concentrators, ports and customers.

Conventional optimization approaches and solution methodologies simplify the optimization of mining complexes into separate, sequential, linear optimization steps, leading to the sub-optimal performance of the value chain as a whole. Importantly, the existing models ignore the uncertainty in material supply and the compounded negative effects it has on the performance of the downstream operations and forecasts generated with respect to production and financial valuations. Similarly, they are limited in their forecasting capabilities by the estimated models representing the mineral deposits used as input. The latter models misrepresent (reduce) the variability of the deposit attributes of interest, and also misrepresent the proportions of both low grade materials (over represented) and high grade materials (under represented). For descriptions and implementations of conventional mine planning methods please refer to Hustrulid et al. (2006).

The simultaneous stochastic optimization of mining complexes presented in the following sections address the above limitations by modelling and optimizing all components of the mining complex in a single mathematical model and capitalizes on their synergies. A major aspect of the simultaneous stochastic optimization presented herein is that, as noted above, it includes geological uncertainty so as to manage the related risk. This type of uncertainty is quantified and accounted for through the use of a group of geostatistically (stochastically) simulated scenarios of all pertinent attributes of the mineral deposits in the mining complex. In addition, and unlike conventional approaches, simulated scenarios represent the overall variability and proportions of the materials expected to flow through the mineral value chain, given all available information. A set of simulated scenarios of the pertinent attributes of a mineral deposit replaces the well-established conventional practice of using a single estimated (average type) model of a deposit (David 1977). This set of scenarios is integrated into the optimization process by a corresponding stochastic mathematical programming formulation. The output of such an optimization formulation generates a single optimal production schedule for the mines involved, as well as the risk management and assessment for all related forecasts of key performance indicators.

In general, existing attempts to optimize mining complexes require major simplifications in order to provide a linear optimization model that can be solved in a reasonable amount of time. The most frequent simplification is to optimize a mine independently of others within an operation, and to decompose its production schedule optimization into a series of sequential steps (Dagdelen 2007), which include the delineation of the final (ultimate) pit limit (Lerchs and Grossmann 1965; Picard 1976), pushback design (Ramazan and Dagdelen 1998), extraction sequencing within the pushbacks (Johnson 1968; Dagdelen 1985; Albor Consuegra and Dimitrakopoulos 2010) and, finally, cut-off grade and stockpile optimization (Lane 1988; Asad and Topal 2011; Rendu 2014). Another major limitation of a sequential approach is the use of economic values for the mining blocks representing a mineral deposit, hence pre-determined destinations are assigned to mining blocks prior to defining the ultimate pit and pushbacks.

To improve this sequential framework that is typically used in established practices, other optimization models focus on mining block extraction sequencing (Caccetta and Hill 2003). Due to the large number of integer decision variables that are used to decide the optimal extraction period for each individual block, research has investigated more efficient ways to solve these models (Ramazan and Dimitrakopoulos 2004; Bienstock and Zuckerberg 2010; Bley et al. 2012; Lambert and Newman 2013; others). As noted above, these existing models, however, ignore one or several aspects of real-world mining complexes that are required to provide a representative model of a mining operation, such as post-extraction mining block destination decisions, stockpiling decisions and non-linear interactions that occur in processing streams.

Approaches to the simultaneous or global optimization of mining complexes to date are limited. These approaches often only optimize specific components of a mining complex, and do not account for uncertainties, consider predefined mine production schedules, and assess processing capacity and material flow (Whittle 2010). Hoerger et al. (1999) formulated a model for optimizing a gold mining complex with multiple mines with given production schedules, stockpiles and processing facilities, to show how their approach improves performance. Stone et al. (2007) show similar efforts based on a different mathematical model. Dagdelen and Traore (2014) show a combination of open pit and underground gold mines that are very far from the processing plant, thus, considering the transportation of materials is critical. Another challenge associated with the (non-stochastic) global optimization of mining complexes, including the above examples, stems from the non-linearity that arises from integrating stockpiling, blending and non-linear transformations that occur in the various processing streams (e.g., grade-recovery curves and throughput-hardness relationships). To circumvent these challenges, optimization models such as the ones discussed above, are simplified in order to obtain a linear formulation. Noted above, the most common example of these simplifications is the use of an economic value per mining block in a mineral deposit in the optimization process since the start of optimization methods for mining applications to today (Lerchs and Grossmann 1965; Johnson 1968; Picard 1976; Dagdelen 1985; Caccetta and Hill 2003; Hustrulid et al. 2006; Stone et al. 2007; Whittle 2010; Lambert and Newman 2013; Singh et al. 2014; others). By calculating the economic value of a mining block independently of others, the optimization model ignores the ability to extract, blend and process materials that would improve the performance of the mining complex as a whole. The simultaneous stochastic optimization of a mineral value chain presented herein considers the economic value of the products sold, thus, overcoming the limitations of assigning economic values of individual blocks and optimizing based on these values. This new approach to modelling permits the incorporation of non-linear interactions of materials in the processing streams.

Concerns for conventional optimization of mine plans and production schedules with respect to the use of single estimated models of orebodies (Ravenscroft 1992; Dowd 1994; Dimitrakopoulos et al. 2002) led to new optimization models based on stochastic integer programming (Birge and Louveaux 2011) for integrating and managing geological uncertainty directly into the optimization of mining operations. Ramazan and Dimitrakopoulos (2005, 2013) proposed a two-stage stochastic integer program (SIP) generating a long-term production schedule that maximizes the net present value (NPV) of the materials mined and, at the same time, minimizes the risk of not meeting production targets, such as ore, total material movement and metal production. This basic model has been tested in several case studies reviewed in Dimitrakopoulos (2011), and has been extended to incorporate more complex aspects (Kumral 2010; Benndorf and Dimitrakopoulos 2013; Dimitrakopoulos and Jewbali 2013; Leite and Dimitrakopoulos 2014; Carpentier et al. 2015; Gemcom 2016). While the above approach outperforms deterministic optimization methods, it is limited by (1) only considering operations with a single mine; (2) assuming an a priori definition of ore and waste material through a predefined cut-off grade policy similarly to the conventional approaches and, hence, does not dynamically optimize where materials are sent post-extraction; and (3) it does not optimize the downstream processes. SIP methods that integrate destination decisions are available; Boland et al. (2008) decided destinations of mining blocks for each scenario; Montiel and Dimitrakopoulos (2013) decided where each block is sent, regardless of the scenario; and Menabde et al. (2007) used a robust cut-off grade approach where mining blocks with similar grades were sent. More recently, simultaneous stochastic optimization of mineral value chains similar to the one presented here have been developed (Montiel and Dimitrakopoulos 2015; Goodfellow and Dimitrakopoulos 2016). Related is also the work by Pimentel et al. (2013), Singh et al. (2014) and methods in Méndez et al. (2006).

In the following sections, the proposed stochastic integer programming model is first detailed and solution methods discussed. Next, two applications of the proposed method are presented, one at nickel laterite operation and the second at a copper–gold mining complex. Finally, conclusions are presented.

## A Mathematical Model for the Simultaneous Stochastic Optimization of Mineral Value Chains

### Concepts and Definitions

The model for mining complexes may vary, depending on the type of commodity produced, as well as geographical and geological conditions. It is, therefore, difficult to create a specific model that accommodates all possible mining operations. A generic modelling approach is discussed herein, and may be adapted to model the unique intricacies of any operation, as may be needed. This section provides a foundation by outlining basic concepts and definitions for a generalized modelling approach. The term “material” describes a product that is extracted from a mine or generated via blending, separation or processing. Often, these materials have unique mineralogical or geometallurgical characteristics that influence the decision for where it can be sent for further blending or treatment in a processing stream or waste. An “attribute” is a term used to describe a property or characteristic of a material of interest, and may be categorized into two groups. Primary attributes $(p\in \mathbb {P})$ are the variables of interest that are sent from one location in the value chain to another, such as metal tonnages, or total tonnage. The values of primary attributes may be added together directly, that is, adding total tonnages for material received from a group of mines. Hereditary attributes $(h\in \mathbb {H})$ are the variables of interest at specific locations in the value chain that are of interest, but are not necessarily forwarded between locations in the value chain. Examples include mining, stockpiling and processing costs, revenues from metal sale, throughput rates, energy consumption and revenues from the sale of the product. These attributes are calculated using (non-) linear equations, $f_h \left( {p,i} \right)$, which need to be defined per case and are evaluated dynamically during optimization.

A mineral value chain, $${\mathcal {C}}$$, may include sets of mines $$({\mathcal {M}})$$, stockpiles $$({\mathcal {S}})$$ or other destinations $$({\mathcal {D}})$$, that is, $${\mathcal {C}}={\mathcal {M}}\cup {\mathcal {S}}\cup {\mathcal {D}}$$. To accommodate the description of the modelling and optimization methods developed herein, consider the case where each location in the mining complex may receive products from multiple sources, but generates only a single product. The more general case where multiple products are generated is a relatively minor extension. Only geological uncertainty is considered in this work, whereby each block $$b\in \mathbb {B}_m $$ at mine $$m\in {\mathcal {M}}$$ has simulated attributes and material types. A set of equally probable scenarios, $$\mathbb {S}$$, describes the combinations of the various simulations from independent sources of uncertainty. For example, if the mining complex is comprised of two mines, each being characterized by 20 geological simulations, there are 400 scenarios in $$\mathbb {S}$$. The value of block *b*’s primary attribute $$(p\in \mathbb {P})$$ for each scenario $$(s\in \mathbb {S})$$ is denoted by $$\beta _{p,b,s}$$. The set of locations in the mining complex that send material to a location $$i\in {\mathcal {S}}\cup {\mathcal {D}}$$ is denoted by $${\mathcal {J}}\left( i \right) $$, i.e., the set of locations that are incoming at *i*. Alternatively, the set of locations that receive materials from $$i\in {\mathcal {C}}$$ is denoted by $${\mathcal {O}}\left( i \right) $$, i.e., the set of locations that are outgoing from *i*. The value of a primary or hereditary attribute in a given scenario $$s\in \mathbb {S}$$ and time period $$t\in \mathbb {T}$$ at a location $$i\in {\mathcal {C}}$$ is denoted by the variables $$v_{p,i,t,s} $$ and $$v_{h,i,t,s}$$, respectively. Similarly, the recovery of a primary attribute is given by the variable $$r_{p,i,t,s} $$, which is either a constant factor, or equal to the value of a hereditary attribute, which may, for example, be governed by a grade-recovery curve (i.e., $$r_{p,i,t,s} =v_{h,i,t,s} =f_h \left( {p,i} \right) $$).

The previous terms are used in the next section to develop a model that defines where materials are sent in a mining complex, and the (potentially non-linear) transformations that occur at each location. To define the quantities of the attributes that flow through the mining complex, it is necessary to define the three types of decision variables that the stochastic simultaneous optimizer can modify:

**Fig. 2:**
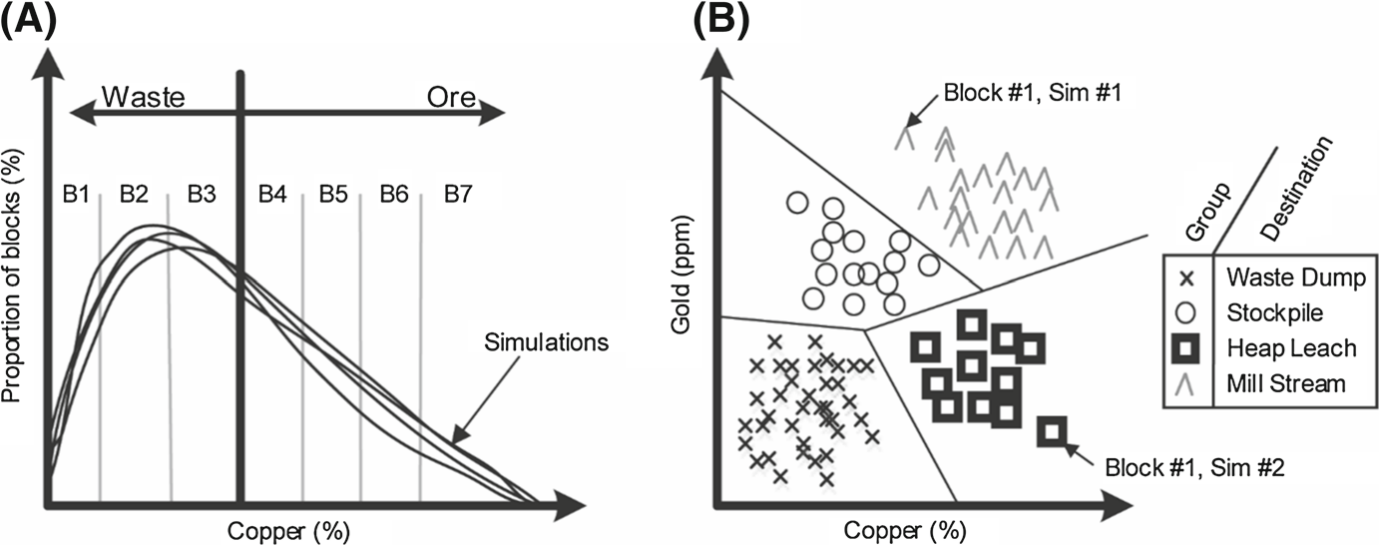
**a** Robust cut-off grades based on “bins”. **b** Extension to create destination policies based on multivariate distributions of primary attributes (e.g., copper and gold grades). Note that a block’s destination may change between simulations according to its simulated attributes

- Production scheduling decisions $(x_{b,t} \in \left\{ {0,1} \right\} )$ define whether (1) or not (0) a block $b\in \mathbb {B}_m$ from mine $m\in {\mathcal {M}}$ is extracted in period $t\in \mathbb {T}$. It is noted that in order to safely extract a mining block *b*, it is necessary to have first extracted all blocks in its set of overlying blocks, $\mathbb {O}\left( b \right)$.
- Destination policy decisions $(z_{g,j,t} \in \left\{ {0,1} \right\} )$ define whether (1) or not (0) a group $(g\in \mathbb {G})$ of a material is sent to destination $j\in {\mathcal {O}}\left( g \right)$ in period $t\in \mathbb {T}$. These policies are an extension of the robust cut-off grade policies developed by Menabde et al. (2007), but consider multivariate distributions. Figure 2a compares the single-element approach proposed by Menabde et al. (2007) with the multi-element approach adopted herein. In the single-element case (A), the distribution of grades is discretized into grade “bins”, and the optimizer is tasked with finding the minimum grade bin from which all bins above are processed as ore material (i.e., the cut-off grade). Cut-off grade decisions are not ideal for mining complexes with multiple elements because they ignore correlations between variables. For the example of a nickel laterite mining complex in Sect. 3.1, a decision based solely on high nickel grades will result in sending materials with low quantities of magnesia. This can have a detrimental impact on the performance of the processing plant due to a constraint on the silica-to-magnesia ratio of the treated products. In the proposed approach, Fig. 2b, groups (bins) are generated using a clustering algorithm based on multiple elements (e.g., grades) within each material type. The optimizer is tasked with deciding where each group (cluster) is sent in each period. As a result of having decision variables based on these groups that are functions of multiple variables, the destination decisions are more adept at creating destination policies for mining complexes with multiple elements and blending constraints in the processing streams. In the example of a nickel laterite operation, where the destination policies are based on groups defined by nickel, silica and magnesia grades, the optimizer has the ability to blend material with a high nickel grade and a low silica-to-magnesia ratio with material with low-grade nickel and a higher silica-to-magnesia ratio. This can lead to a homogenous product that satisfies the quality constraints on the secondary elements at the processor. These groups may be generated using a pre-processing step with the k-means++ clustering algorithm (Lloyd 1982; Arthur and Vassilvitskii 2007), whereby the number of groups for each material type are defined a priori, and clustering is performed based on the block’s primary attributes, $\beta _{p,b,s}$. In this pre-processing step, a parameter, $\theta _{b,g,s} \in \left\{ {0,1} \right\}$ is generated to define whether (1) or not (0) block $b\in \mathbb {B}_m$ belongs to the group $g\in \mathbb {G}$ in scenario $s\in \mathbb {S}$. It is important to note that when a block’s material type and attributes are simulated, a block may be sent to different destinations across the various scenarios. However, the destinations are optimized using an over-arching destination policy based on the groups, which is scenario-independent.
- Processing stream decisions $(y_{i,j,t,s} \in \left[ {0,1} \right] )$ define the proportion of a product sent from a location $i\in {\mathcal {S}}\cup {\mathcal {D}}$ to a destination $j\in {\mathcal {O}}\left( i \right)$. It is noted that, unlike the previous two decision variables, these variables are scenario-dependent decisions, which may, for example, be used to define the quantity of material processed from a stockpile, if there happens to be a shortfall in the quantity of ore material sent directly from the mines.

### A Two-Stage Stochastic Optimization Model

A two-stage stochastic integer programming model (SIP), as per Birge and Louveaux (2011), is formulated to generate a life-of-mine (LOM) production schedule, destination policies and the use of the available processing streams. Given the considerations in the previous section, a general optimization model is presented, which can be adopted to different cases. Similar to the SIP defined by Ramazan and Dimitrakopoulos (2005, 2013), the primary objective is to maximize the discounted net present value, while simultaneously managing risk by accounting for and minimizing deviations from the various constraints in the value chain model. The model focuses primarily on the simultaneous stochastic optimization of a mining complex under geological uncertainty, however, it can be extended to integrate other sources of uncertainty, such as mine and processing capacities and grade-recovery relationships.

*Inputs and parameters:*

- Simulated block attributes, $\beta _{p,b,s}$.
- Block extraction precedence relationships, $\mathbb {O}\left( b \right)$, e.g. Khalokakaie et al. (2000).
- Block group memberships, $\theta _{b,g,s}$.
- A model of the mining complex, i.e. ${\mathcal {O}}\left( i \right)$ and ${\mathcal {J}}\left( i \right) \,\forall i\in {\mathcal {S}}\cup {\mathcal {D}}\cup \mathbb {G}$.
- A model of the hereditary attribute transformation functions, $f_h \left( {p,i} \right)$.
- Time-discounted price (or cost) per unit of attribute, $$p_{h,i,t} $$. Often, this is only a discount rate, and is used to calculate the net present value.
- Upper- and lower-bounds for an attribute, $$U_{h,i,t} $$ and $$L_{h,i,t} $$, respectively. Often, these will be required for tonnage, metal production and product quality constraints, but may be used to identify any potential bottleneck in the mineral value chain.
- Penalty costs, $$c_{h,i,t}^+$$ and $$c_{h,i,t}^- $$, which are used to penalize deviations from the upper- and lower-bounds. These penalty costs may be time-varied to provide geological risk discounting, i.e. $$c_{h,i,t} =c_{h,i} /\left( 1+grd_{h,i}\right) ^{t}$$, where $$c_{h,i} $$ is a base penalty cost and $$grd_{h,i} $$ is the geological risk discount rate for the attribute of interest (*h*). For further discussion of this parameter, see Benndorf and Dimitrakopoulos (2013).

*Objective function*

1 $$\begin{aligned} \max \frac{1}{\left| \mathbb {S} \right| }\underbrace{\sum _{s\in \mathbb {S}} \sum _{t\in \mathbb {T}} \sum _{h\in \mathbb {H}} p_{h,i,t} \cdot v_{h,i,t,s}}_{\mathrm{Discounted\,costs\,and\,revenues}} -\frac{1}{\left| \mathbb {S} \right| }\underbrace{\sum _{s\in \mathbb {S}} \sum _{t\in \mathbb {T}} \sum _{h\in \mathbb {H}} c_{h,i,t}^+ \cdot d_{h,i,t,s}^+ +c_{h,i,t}^- \cdot d_{h,i,t,s}^- }_{\mathrm{Penalties \,for \,deviations \,from \,targets}}. \end{aligned}$$

*Subject to*

Mine’s reserves and slope constraints, which enforce slope stability and one-time extraction of blocks.

2 $$\begin{aligned} \sum _{t\in \mathbb {T}} x_{b,t}\le & {} 1\quad \forall b \in \mathbb {B}_m ,\,m\in {\mathcal {M}}, \nonumber \\ x_{b,t}\le & {} \sum _{t^{\prime }=1}^{t}x_{u,t^{\prime }} \quad \forall b\in \mathbb {B}_{m},m\in \mathcal {M},\,u\in \mathbb {O}(b),\,t\in \mathbb {T}. \end{aligned}$$

Destination policy constraints, which ensure a group of material is sent to a single destination.

3 $$\begin{aligned} \sum _{j\in {\mathcal {O}}\left( g \right) } z_{g,j,t} =1\quad \forall g\in \mathbb {G},\,t\in \mathbb {T}. \end{aligned}$$

Processing stream constraints, which calculate the quantity of primary attributes at each location and ensure mass-balancing.

4 $$\begin{aligned} v_{p,j,\left( {t+1} \right) ,s}= & {} \underbrace{v_{p,j,t,s} \cdot \left( {1-\sum _{k\in {\mathcal {O}}\left( j \right) } y_{j,k,t,s} } \right) }_{\mathrm{Material \,from \,previous \,period}} +\underbrace{\sum _{i\in {\mathcal {J}}\left( j \right) \backslash \mathbb {G}} r_{p,i,t,s} \cdot v_{p,i,t,s} \cdot y_{i,j,t,s} }_{\mathrm{Incoming\,materials\,from\,other\,locations}} \nonumber \\&+\underbrace{\sum _{g\in {\mathcal {J}}\left( j \right) \cap \mathbb {G}} \left( {\sum _{b\in \mathbb {B}_m } \sum _{m\in {\mathcal {M}}} \theta _{b,g,s} \cdot \beta _{p,b,s} \cdot x_{b,\left( {t+1} \right) } } \right) \cdot z_{g,j,\left( {t+1} \right) } }_{\mathrm{Materials\,sent\,directly\,from\,mines}} \nonumber \\&\forall p\in \mathbb {P},j\in {\mathcal {S}}\cup {\mathcal {D}},\,t\in \mathbb {T},\,s\in \mathbb {S}, \nonumber \\&\sum _{j\in {\mathcal {O}}\left( i \right) } y_{i,j,t,s} =1\quad \forall i\in {\mathcal {D}},t\in \mathbb {T},s\in \mathbb {S} \nonumber \\&\sum _{j\in {\mathcal {O}}\left( i \right) } y_{i,j,t,s} \le 1\quad \forall i\in {\mathcal {S}},t\in \mathbb {T},s\in \mathbb {S}. \end{aligned}$$

Attribute calculations—used to calculate the values of the hereditary attributes based on the values of the primary attributes.

5 $$\begin{aligned} v_{h,i,t,s}= & {} f_h \left( {p,i} \right) \,\,\forall h\in \mathbb {H},i\in {\mathcal {S}}\cup {\mathcal {D}}\cup {\mathcal {M}},\,t\in \mathbb {T},\,s\in \mathbb {S} \nonumber \\ v_{p,m,t,s}= & {} \sum _{b\in \mathbb {B}_m } \beta _{p,b,s} \cdot x_{b,t} \quad \forall m\in {\mathcal {M}},\,p\in \mathbb {P},t\in \mathbb {T},s\in \mathbb {S}. \end{aligned}$$

Deviation constraints—calculate the amount of constraint violation from upper- and lower-bounds imposed on hereditary attributes.

6 $$\begin{aligned} v_{h,i,t,s} -d_{h,i,t,s}^+\le & {} U_{h,i,t} \quad \forall h\in \mathbb {H},\,t\in \mathbb {T},\,s\in \mathbb {S} \nonumber \\ v_{h,i,t,s} +d_{h,i,t,s}^-\ge & {} L_{h,i,t} \quad \forall h\in \mathbb {H},\,t\in \mathbb {T},\,s\in \mathbb {S}. \end{aligned}$$

Recovery calculations.

7 $$\begin{aligned} r_{p,i,t,s}= & {} 1\quad \forall p\in \mathbb {P},i\in {\mathcal {S}},t\in \mathbb {T},s\in \mathbb {S} \nonumber \\ r_{p,i,t,s}= & {} v_{h,i,t,s} \quad \forall p\in \mathbb {P},i\in {\mathcal {D}},\,t\in \mathbb {T},\,s\in \mathbb {S}. \end{aligned}$$

End-of-year stockpile quantity calculations (optional).

8 $$\begin{aligned} v_{h,i,t,s} =v_{p,i,t,s} \cdot \left( {1-\mathop \sum \limits _{i\in {\mathcal {O}}\left( i \right) } y_{i,j,t,s} } \right) \quad \forall h\in \mathbb {H},i\in {\mathcal {S}},t\in \mathbb {T},s\in \mathbb {S}. \end{aligned}$$

Variable definitions.

9 $$\begin{aligned}&x_{b,t} \in \left\{ {0,1} \right\} \quad \forall b\in \mathbb {B}_m ,m\in {\mathcal {M}},t\in \mathbb {T} \nonumber \\&\quad z_{g,j,t} \in \left\{ {0,1} \right\} \quad \forall g\in \mathbb {G},\,j\in {\mathcal {O}}\left( g \right) ,t\in \mathbb {T} \nonumber \\&\quad y_{i,j,t,s} \in \left[ {0,1} \right] \quad \forall i\in {\mathcal {S}}\cup {\mathcal {D}},j\in {\mathcal {O}}\left( i \right) ,t\in \mathbb {T},\,s\in \mathbb {S} \nonumber \\&\quad v_{p,i,t,s} \ge 0\quad \forall p\in \mathbb {P},i\in {\mathcal {S}}\cup {\mathcal {D}}\cup {\mathcal {M}},\,t\in \mathbb {T},s\in \mathbb {S} \nonumber \\&\quad v_{h,i,t,s} \in \mathbb {R}\quad \forall h\in \mathbb {H},i\in {\mathcal {S}}\cup {\mathcal {D}}\cup {\mathcal {M}},\,t\in \mathbb {T},s\in \mathbb {S} \nonumber \\&\quad r_{p,i,t,s} \in \left[ {0,1} \right] \,\,\forall p\in \mathbb {P},\,i\in {\mathcal {S}}\cup {\mathcal {D}},t\in \mathbb {T},s\in \mathbb {S} \nonumber \\&\quad d_{h,i,t,s}^+ ,\,d_{h,i,t,s}^- \ge 0\quad \forall h\in \mathbb {H},i\in {\mathcal {S}}\cup {\mathcal {D}}\cup {\mathcal {M}},t\in \mathbb {T},s\in \mathbb {S}. \end{aligned}$$

Given the possibility of using stockpiles and incorporate transformation functions (e.g., grade-recovery curves), traditional mathematical optimizers are generally unable to optimize over these non-linear aspects, particularly for large-scale and real-world examples. As a result, a solver has been developed that uses a combination of metaheuristic algorithms to obtain solutions. Metaheuristics are algorithmic optimizers that do not necessarily provide a mathematically optimal solution, but are adaptable for various types of problems, including non-linear optimization models, and have been successfully used in the past for mine design and production scheduling models (Godoy 2003; Lamghari and Dimitrakopoulos 2012; Goodfellow and Dimitrakopoulos 2013; Lamghari et al. 2014).

The approach discussed herein uses two metaheuristics in an iterative manner to help avoid being trapped in local optima due to the large number of decision variables that may be modelled for realistic-sized models. A variation of the simulated annealing algorithm (Kirkpatrick et al. 1983; Geman and Geman 1984) is first used to optimize the multi-mine production scheduling, destination policy and processing stream decision variables. One of the major challenges of simulated annealing used for the simultaneous optimization model is the large number of scenario-dependent processing stream decisions; it becomes computationally prohibitive to modify a single variable and evaluate the impacts of all downstream decisions. As a result, once the simulated annealing algorithm performs a specified number of iterations or is determined to be trapped in a local optimum, a second population-based metaheuristic is used for local improvement. Specifically, the particle swarm optimization algorithm (Kennedy and Eberhart 1995; Poli et al. 2007) or differential evolution (Storn and Price 1997; Price et al. 2005) are used to optimize the downstream (post-extraction) destination policy and processing stream decision variables simultaneously. Unlike the solution perturbation mechanisms used in the simulated annealing algorithm, which only modify a single class of decision variables at each iteration, these two metaheuristics are capable of modifying all downstream variables simultaneously. For a full overview of the algorithm employed, and a comparison of the algorithm’s performance with the different population-based metaheuristics on the downstream decision variables, the reader is referred to Goodfellow and Dimitrakopoulos (2016), with the note that this approach may be used in different application, such as the optimization of large-scale oil field operations (Bellout et al. 2012; Isebor et al. 2014). The combination of the metaheuristics has demonstrated the ability to improve upon existing solutions, thus, helping to prevent the solver from being trapped in local optima. The following section demonstrates the effectiveness of the simultaneous stochastic optimization model and solver. In the first application, which focuses on downstream optimization for a blending problem, the solution is generated using the simulated annealing algorithm with particle swarm optimization, which was deemed sufficient to obtain a consistent, high-quality solution. In the second application, which introduces extraction sequencing variables, results are shown for the solution generated using simulated annealing and differential evolution, which, through experimentation, was determined to produce better and more consistent solutions for a wide variety of larger optimization models.

**Fig. 3:**
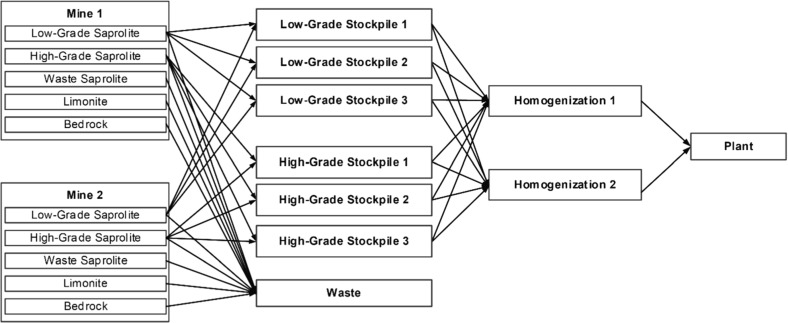
Material flow diagram for the nickel laterite value chain

## Applications, Benchmarking and Analysis

### Blending Policy for a Nickel Laterite Mineral Value Chain

The flow of materials through a nickel laterite mineral value chain is shown in Fig. 3. The purpose of this application is to highlight the importance and the substantial value added by integrating geological uncertainty into destination policy optimization. The optimizer seeks to generate an optimal definition of a multi-element destination policy (based on nickel, iron, silica and magnesia grades, and a dry tonnage density factor), and the use of the stockpiles and homogenization piles. It is noted that production scheduling is not performed in this application; the production schedule used is based on an existing plan. Using the generalized modelling methodology, it is possible to model the flow of the materials from the two mines to the processing plant. Rather than presenting the entire mathematical model, the general goals for the optimizer are listed in order of importance, as follows:

1. Maximize NPV.
2. Satisfy the plant feed’s silica-to-magnesia ratio $(\hbox {SiO}_{2}{:}\hbox {MgO})$, which should be between 1.5 and 1.8.
3. Meet the plant’s production target.
4. Satisfy plant feed iron grade blending constraints, which should be between 12 and 16%.
5. Satisfy stockpile capacity constraints.

The estimated orebody models for the two mineral deposits have been provided by the mine and were generated using ordinary kriging (David 1977). Twenty geological simulations have been generated using the direct block min/max autocorrelation factor simulation method (Boucher and Dimitrakopoulos 2009), which results in 400 scenarios in total. First, the limonite and saprolite layer thicknesses are jointly simulated. The primary attributes (nickel, silica, magnesia, iron and dry tonnage factor) are simulated within the saprolite layer for each of the lithological simulations. It is noted that, while nickel laterite deposits are notoriously variable in their geological conditions, only twenty simulations are required in this study to characterize the geological uncertainty from each of the deposits at the mine’s production scale. The number of scenarios that is required can be determined experimentally by gradually adding scenarios, and evaluating the quality and robustness of the resulting optimized solution with a second set of simulations not used during the optimization process. This result coincides with a study presented by Albor Consuegra and Dimitrakopoulos (2009), which demonstrates that 15 simulations can be used to generate stable LOM production schedules. This phenomenon is expected because a production year for a mine typically includes several thousand mining blocks; this is often referred to as the support-scale effects or volume-variance relationship.

**Fig. 4:**
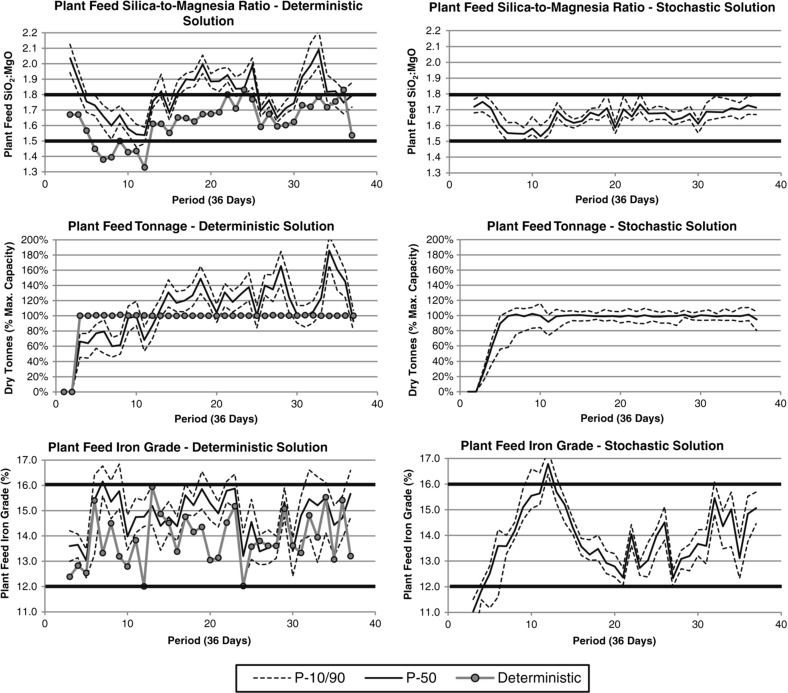
Comparison of deterministic and stochastic risk profiles for the chemistry and tonnage at the processing plant

Using the estimated orebody model, a deterministic optimization is performed using the proposed methods. Figure 4 (left) shows a summary of the $\hbox {SiO}_{2}{:}\hbox {MgO}$, tonnage and iron grades for the material received at the processing plant from the homogenization piles. Generally, the optimizer is able to satisfy the key quality constraints on the $\hbox {SiO}_{2}{:}\hbox {MgO}$ and the iron grade, and is able to fill the processing plant up to capacity over time. Using the set of orebody simulations, it is possible to perform a sensitivity analysis of the destination policies generated by freezing these decision variables, $z_{g,j,t}$, which were generated using the deterministic-equivalent optimizer, and re-optimizing the processing stream (stockpile and homogenization pile) decision variables for each of the scenarios. The results are summarized on the same figure using a risk profile, which indicates the P-10, P-50 and P-90 exceedance probabilities (i.e., the value for which 10, 50 or 90% of the simulations lie below). While the destination policies generated for the estimated orebody models are able to satisfy the blending and production constraints, the risk analysis indicates that these policies are not adequate when considering the spatial variability and uncertainty in the saprolite layers, along with the variability of the primary attributes of interest. As a result, this destination policy does not provide a feed to the processing plant that satisfies the blended quality constraints, and generally misclassifies ore and waste materials, which causes the plant feed tonnages to be under or over the target tonnage. This is simply a result of the fact that simulations, by construction, better-capture the high- and low-grades of the distributions for the elements, and better-reproduce the spatial (cross-) correlations between the elements (Ni, $\hbox {SiO}_{2}$, MgO) and materials (limonite and saprolite) that are seen in the original data set. It is noted, however, that this result does not relate to the performance of the optimizer or the quality of the solution generated. The risk profiles highlight the need to adopt stochastic approaches when optimizing mining complexes, given variability, both in terms of materials and metal content.

A stochastic optimizer works with all geological simulations simultaneously, and attempts to find a single destination policy, and the scenario-dependent processing stream (stockpile) decision variables. Figure 4 (right) shows a summary of the risk profiles for a stochastic design. It is noted that, unlike the risk profiles from the deterministic design, the stochastic design is able to satisfy the key constraints of interest, namely, the $\hbox {SiO}_{2}{:}\hbox {MgO}$ ratio, iron grade and plant feed tonnage. It is interesting to note that in the first ten periods, there is more variability in tonnage than in the later periods; this is largely attributed to two factors: (1) prioritizing a consistent $\hbox {SiO}_{2}{:}\hbox {MgO}$ ratio over tonnages; and (2) developing the quantities of materials in the stockpiles, which act as a buffer between the highly variable in-situ saprolite material and the material sent to the processing plant. Not only is the stochastic destination policy able to satisfy critical blending constraints, but it is much more practical and realistic. The NPV (not shown for confidentiality purposes) is 3% higher than what the deterministic-equivalent depicts with the estimated orebody model.

**Fig. 5:**
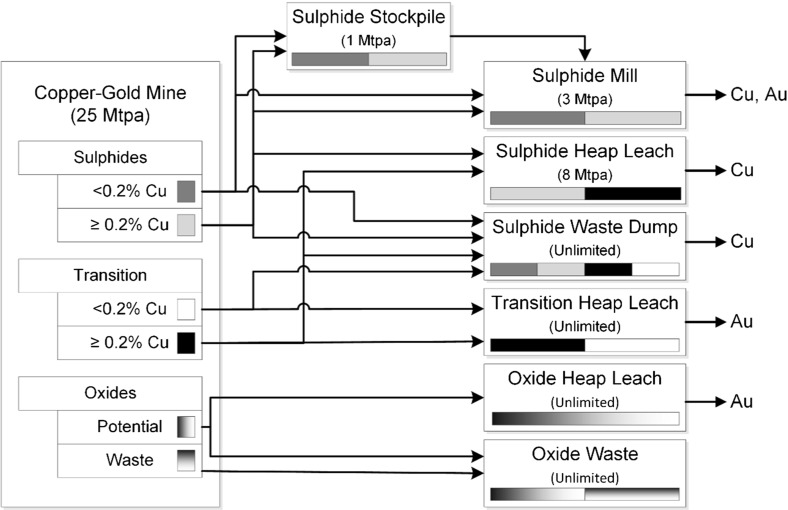
Material flow through the copper–gold mining complex

**Fig. 6:**
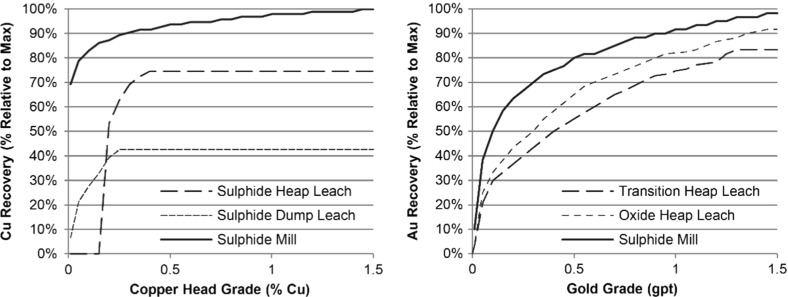
Non-linear grade-recovery curves for copper (*left*) and gold (*right*), based on the head grade from the mine

### Simultaneous Stochastic Optimization at a Copper–Gold Mining Complex

The second application involves the stochastic global optimization of a copper–gold open pit mining complex, which considers simultaneous production scheduling, destination policies and processing stream decisions. Figure 5 provides an overview of the material flows through the mining complex. The key destinations of interest are the sulphide mill, which has a capacity of 3 Mtpa, and the sulphide heap leach, which has a capacity of 8 Mtpa. A stockpile may be used to store additional sulphide material that is sent to the mill. All other locations are considered to have an unlimited capacity. An interesting aspect of this study is the use of non-linear grade-recovery curves for the copper and gold head grades at the respective processing stream (Fig. 6). The use of these downstream grade-recovery relationships is often avoided in existing studies because of the non-linear relationship it introduces to the optimization model. Rather than specifying the recovery for each block in each simulation, which assumes that each block is processed independently, this approach considers the blended feed of all materials received from the mine. The primary objectives of the optimization are defined as follows, in order of importance:

- Maximize NPV.
- Meet sulphide mill production target (3 Mtpa) and minimize associated risk.
- Meet sulphide heap leach production target (8 Mtpa) and minimize associated risk.
- Obey mine production capacity constraint (25 Mtpa).
- Obey end-of-year stockpile capacity constraints (1 Mtpa).

A set of 35 simulations of the orebody have been generated to represent the deposit and quantify the related geological uncertainty for the simultaneous stochastic optimizer presented in Sect. 2. The solution from the simultaneous stochastic optimizer is next compared with (1) a solution from a commonly used commercial software, and (2) the solution from the deterministic “equivalent” of the method presented in Sect. 2; comparisons highlight the benefits of the proposed method. Note that in absence of an estimated orebody model, an E-type (expected value) orebody model is generated by averaging the grades across all simulations available for each mining block of the deposit; this then serves as input to the deterministic optimization approaches used for comparisons. It should also be noted that a solution generated from an optimization process is referred to as “design”.

**Fig. 7:**
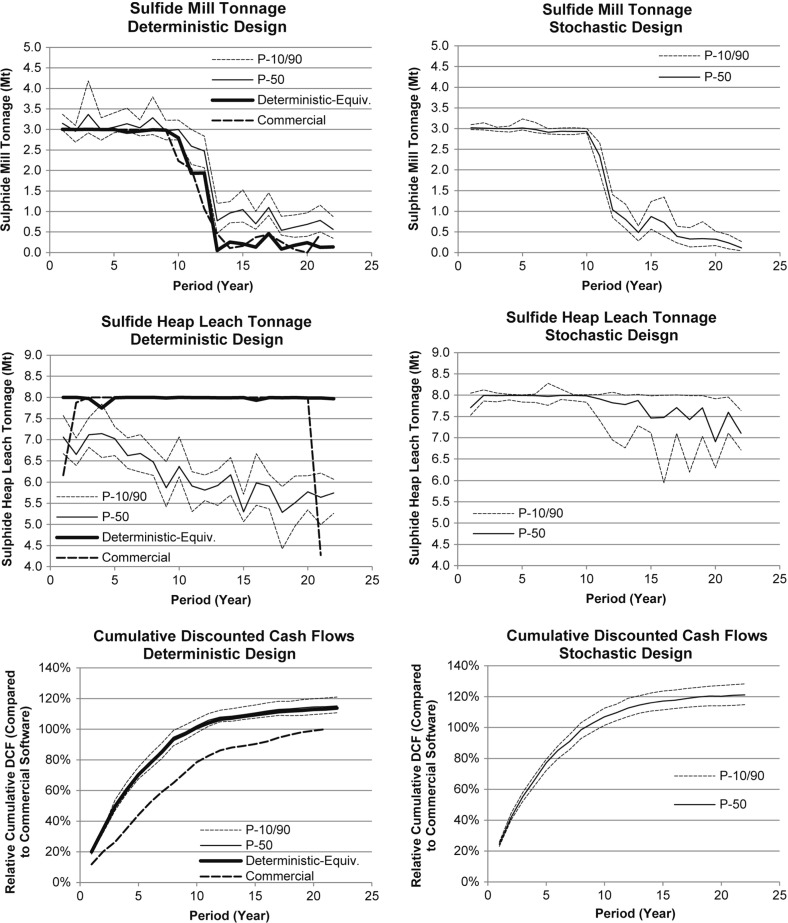
Comparison of risk profiles for the deterministic designs and the stochastic design for the copper–gold mining complex

A widely available commercial mine planning optimization package, is used to generate the “commercial design” and is useful as a basis of comparison for the performance of a conventional sequential optimization framework, as practiced in the industry. The “deterministic-equivalent” design is generated using the deterministic version of the method presented here. Both the commercial and deterministic-equivalent designs are then used to benchmark and demonstrate the advantages of the simultaneous stochastic optimization approach proposed. Figure 7 (left) shows a comparison of the key performance indicators, namely sulphide mill and sulphide heap leach tonnages and the cumulative NPV, for both the commercial and deterministic-equivalent designs, along with the risk profiles for the deterministic-equivalent design. First, it is noted that the deterministic-equivalent design has an additional year of mining, and is better able to meet the sulphide mill and heap leach production targets. This additional year of production is a direct result of the fact that the commercial design employs a sequential optimization framework (ultimate pit, pushback design, production schedule, cut-off grade and stockpile optimization), as discussed in a previous section. The simultaneous stochastic optimization approach is able to extend the life of the operation by 1 year, emphasizing the benefit of using a simultaneous optimizer over a sequential framework. As a result of this, the deterministic-equivalent design has a 13.8% higher NPV than the design generated using commercial software. However, when testing the deterministic-equivalent design with a set of geological simulations, the risk profiles indicate substantial differences and risk related to meeting production targets at both destinations. Despite these differences, the risk profiles for the deterministic-equivalent indicate a similar NPV as the deterministic-equivalent model indicates, which can be attributed to two factors. Firstly, the tonnage processed at the sulphide mill is slightly higher, particularly after year 12. Secondly, and more importantly, the similar NPV is caused by the fact that estimated models (i.e., the E-type model used in this study) smooth out both high- and low-grades, as mentioned earlier. In this study, the ability to characterize the quantity of metal above the cut-off grade appears to have a significant impact on the assessment of the financial performance for the mining complex, particularly in the sulphide mill processing stream, where both copper and gold are generated as products.

The simultaneous stochastic optimizer generates a single LOM production schedule, destination policy and optimizes the use of the stockpile using all simulations. Figure 7 (right) shows the risk profiles of this stochastic design. Unlike the risk profiles from the deterministic-equivalent design (Fig. 7, left), it is apparent that the stochastic design is better able to meet the production targets at the sulphide mill and sulphide heap leach, and simultaneously reduce the risk in terms of the quantities sent. As a result of being able to control ore production, particularly for materials sent to the sulphide heap leach, the NPV of the stochastic design is 6.6% higher than the risk profiles of the deterministic-equivalent design (measured from the P-50 values). Finally, the stochastic design has a 22.6% higher NPV than the commercial solver (measured from the stochastic design’s P-50). These results highlight the importance of not only stochastic optimization for mine design and production scheduling, which has been developed for over a decade, but also highlights the importance of simultaneous stochastic optimization, which seeks to integrate all aspects of decision-making in the mineral value chain in the same optimization model. Despite the conceptual and practical differences from conventional approaches, as well as the knowledge mobilization required to put in regular practice, there is a clear financial benefit to this approach.

## Conclusions

This paper presents the simultaneous stochastic optimization of mining complexes and mineral value chains, and the corresponding two-stage stochastic mixed integer, nonlinear programing formulation. The proposed approach removes limitations of past approaches by (1) integrating and optimizing several parts of a mineral value chain in a single model, and capitalizing on the synergies between various parts of the chain to improve performance. At the same time, the proposed mathematical model (2) integrates quantified geological (metal grades, material types, geometallurgical properties, volumes of materials, and so on) uncertainty and manages the related risk, through the use of geostatistical simulation of the mineral deposits in a mining complex.

To demonstrate the proposed method, two applications are presented. The first deals with the definition of a destination policy for a nickel laterite complex that has multiple stockpiles and blending constraints. The results highlight the fact that ignoring the geological uncertainty related to material and grades can lead to a sub-optimal policy that may lead to severe deviations from product quality requirements. A stochastic optimization approach is able to manage this risk, and generate a blending policy that satisfies stringent constraints. The second application at a copper–gold mining complex integrates life-of-mine production scheduling with destination policies and stockpile management. Comparisons in this application show that the deterministic-equivalent of the proposed simultaneous optimizer is able to generate a design that is 13.8% higher than a design generated using a commercial mine planning tool, which works in a sequential framework. Then, when comparing the risk profiles between the deterministic-equivalent design and the design created from a simultaneous stochastic optimizer, the stochastic approach is consistently better able to meet production targets and manage the associated risk, while simultaneously generating a 6.6% higher NPV than the deterministic-equivalent design. The stochastic approach shows a 22.6% higher NPV than the reported value from the commercial design.

Future work could seek to extend the methods presented herein with more complex downstream aspects and integration of commodity price uncertainties. With respect to other mathematical modelling frameworks, exploring by the optimization of interactions within a mining complex through discrete event simulation—optimization assists modelling smaller-scale interactions important for short-term production planning. Additional areas of interest stemming from the area of smart oil fields are discussed in Benndorf and Jansen (2017) and Lamghari (2017).
